# Hypercalcemia-Driven Spontaneous Tumor Lysis Syndrome in a Case of Recently Diagnosed Multiple Myeloma: An Unusual Clinical Encounter

**DOI:** 10.7759/cureus.69074

**Published:** 2024-09-10

**Authors:** Edgar G Dorsey-Trevino, Taha Al Hassan, Olga G Cantu-Rodriguez

**Affiliations:** 1 Internal Medicine, University of Texas Rio Grande Valley School of Medicine, Weslaco, USA; 2 Hematology, Universidad Autonoma de Nuevo Leon, Monterrey, MEX

**Keywords:** acute spontaneous tumor lysis syndrome, immunoglobulin kappa light chains, malignant hematology, malignant hypercalcemia, newly diagnosed multiple myeloma

## Abstract

Tumor lysis syndrome (TLS) represents an oncological emergency characterized by the rapid disintegration of neoplastic cells and subsequent release of their intracellular content into the systemic circulation due to cytotoxic therapy. However, a rare variant, spontaneous TLS (STLS), can occur without an evident precipitating factor. Prompt recognition of high-risk individuals and initiation of prophylactic interventions are crucial to forestall complications of electrolyte imbalance, such as cardiac arrhythmias or sudden death. We present the case of a 74-year-old male who was referred to the hematologist/oncologist’s office after exhibiting pelvic pain, progressive weakness, fatigue, unintended weight loss over the last five months, and an imaging study revealing osteolytic lesions suggestive of a malignant condition. Initial diagnostic assessment revealed a kappa-light-restricted multiple myeloma, hypercalcemia, hyperuricemia, hyperphosphatemia, and elevated creatinine, diagnosing STLS. This case illustrates an uncommon presentation of STLS and malignant hypercalcemia in the setting of multiple myeloma. We expound upon potential tumor- and patient-specific risk factors previously documented to precipitate STLS, correlate them with our case, and provide comprehensive insights into the diagnostic, therapeutic, and noteworthy educational aspects for clinicians.

## Introduction

The onset of tumor lysis syndrome (TLS) emerges as an oncological emergency, often occurring following induction chemotherapy for hematological malignancies [1]. The predominant pathophysiological process underlying TLS typically involves the cytotoxic impact of chemotherapy on tumor cells and the consequent release of their intracellular content, potentially leading to renal injury, cardiac arrhythmias, and even sudden death [1]. Still, a markedly rare TLS subtype occurs without a cytotoxic agent, that of spontaneous TLS (STLS).

Previous evidence suggests that STLS mortality ranges between 15% and 66%, but this is yet to be well established [2]. While the development of STLS has been reported primarily in acute leukemias or rapidly progressive lymphomas such as Burkitt lymphoma, diffuse large B-cell lymphoma, and lymphoblastic lymphomas [3-5], scarce reports have associated multiple myeloma with STLS. These reports have associated tumor-specific risk factors in the development of STLS [5]. We present a compelling case of STLS triggered by tumor- and patient-specific risk factors and malignant hypercalcemia caused by multiple myeloma.

Through detailed analysis of the patient’s initial clinical presentation, including pertinent laboratory findings, we aim to elucidate the interplay of tumor and patient-specific risk factors contributing to STLS. Our objective is to explore conceivable etiological factors implicated in STLS and correlate them with our case, offering comprehensive insights into diagnostic, therapeutic, and educational aspects for clinicians.

## Case presentation

A 74-year-old male with a past medical history of type II diabetes mellitus, dyslipidemia, and stage IIIb chronic kidney disease complained of lower back pain that started five months ago and an unintended ~14-kilogram weight loss in three months. 

Previous medical reports from another healthcare facility highlighted spinal magnetic resonance imaging that yielded an abnormal signal in the sacrum on the left side at S1, S2, and S3, lateral recess stenosis, and foraminal narrowing at multiple levels, and a subsequent pelvic MRI unveiling heterogeneous neoplastic infiltration of the imaged skeletal bone marrow involving the pelvis, iliac, and femurs. Additionally, the patient's laboratory testing revealed a creatinine of 1.57 mg/dL, uric acid of 12.2 mg/dL, total protein of 16 g/dL, albumin of 3 g/dL, calcium of 11.6 mg/dL (corrected calcium of 12.4 mg/dL), and a parathyroid-stimulating hormone of 13 pg/mL (reference range: 10 - 65 pg/mL). These findings of worsening hypercalcemia, hyperuricemia, and serum creatinine signified end-organ kidney damage, prompting a referral to oncology for further evaluation. 

**Figure 1:**
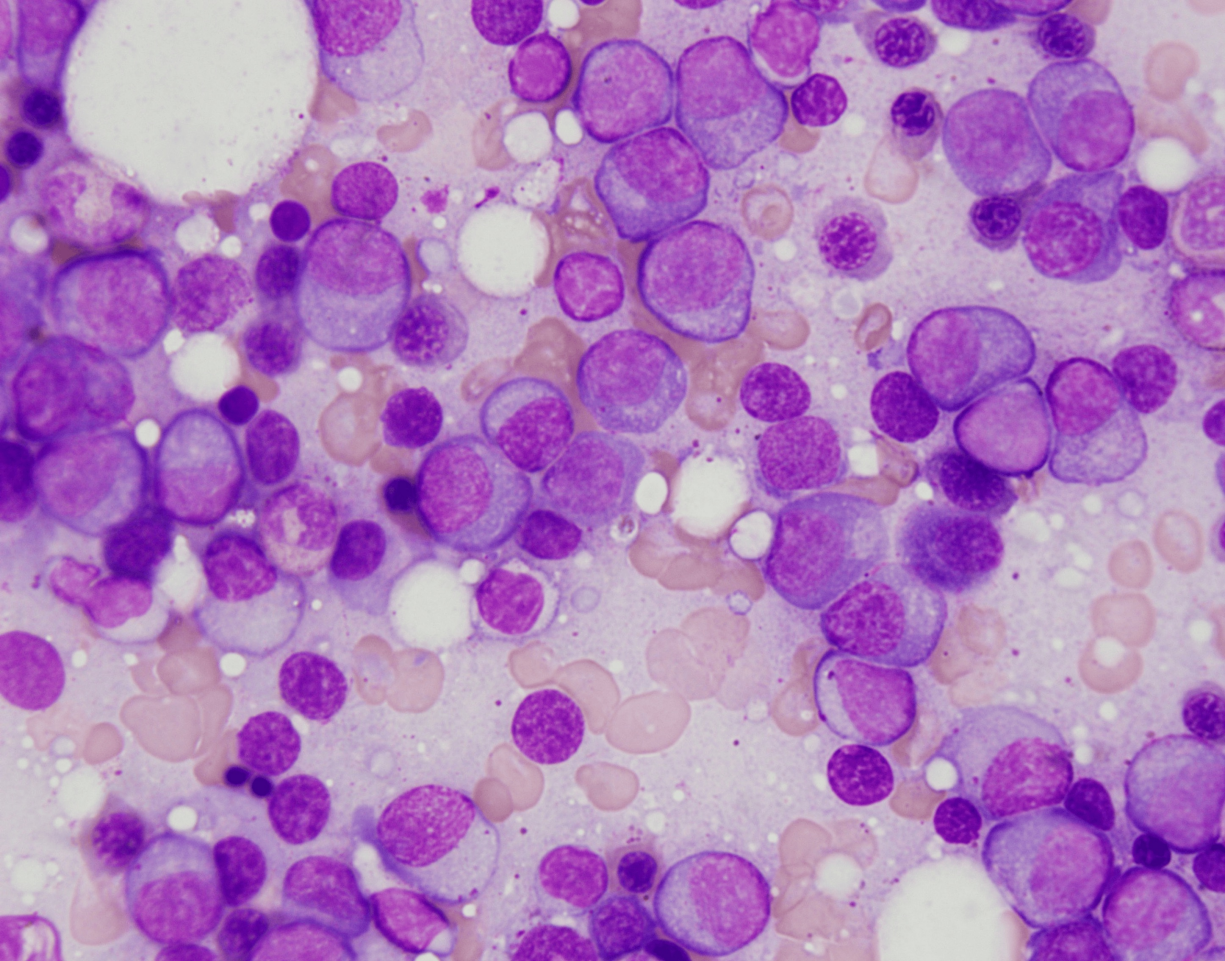
A bone marrow aspirate demonstrates an increased density of clustered plasma cells.

At the oncologist’s office, the patient underwent a workup that included a peripheral blood smear, cytogenetics, and a bone marrow biopsy. The peripheral blood smear demonstrated a normal white blood cell count with atypical plasmacytoid lymphoid cells, microcytic hypochromic anemia with severe anisopoikilocytosis, and rouleaux formation. Cytogenetics revealed a normal male karyotype (46XY) with a 13q deletion/monosomy. Lastly, a bone marrow biopsy revealed hypercellular bone marrow (95% cellularity) with 70-80% light chain kappa-restricted abnormal plasma cells (Figure 1). Based on the International Myeloma Working Group Criteria, the patient was diagnosed with multiple myeloma [6]. Due to his subacute, recent-onset electrolyte abnormalities, the patient was referred to the hospital for medical management.

At the hospital, the patient reported feeling persistent weakness accompanied by tremors, decreased appetite, new-onset ataxia, constipation for the last three days, and reduced energy levels. During the physical examination, the patient’s vital signs were unremarkable. Hypoactive bowel sounds in all quadrants were appreciated. The patient exhibited slightly reduced strength throughout (3/5) of all muscle groups assessed. Reflexes were intact but sluggish throughout. 

Diagnostic assessment

Initial laboratory workup revealed potassium of 3.2 mmol/L, creatinine of 2.9 mg/dL, lactate dehydrogenase of 230 u/L, total protein of 16 g/dL, albumin of 2.4 g/dL, calcium of 12.6 mg/dL (corrected calcium 13.9 mg/dL), phosphate of 5.1 mg/dL, uric acid of 9.1 mg/dL, and a serum osmolarity of 318 mOsm/kg (Table 1). The EKG revealed non-significant T-wave inversion on the inferior leads and a baseline wander on lead V1 (low-voltage artifact). 

**Table 1:** Patient's laboratory values on admission. *Abnormal laboratory result. MCV: mean corpuscular volume; BUN: blood urea nitrogen; MCH: mean corpuscular hemoglobin; RDW: red blood cell distribution width

Parameter	Value	Reference Range
Metabolic Panel		
Sodium (mmol/L)	131*	136 - 145
Potassium (mmol/L)	3.2*	3.5 - 5.3
Chloride (mmol/L)	101	98 - 110
Bicarbonate (mmol/L)	28.9	20 - 31
Calcium (mg/dL)	12.6*	8.6 - 10.4
Total Protein (g/dL)	16*	6.4 - 8.4
Albumin (g/dL)	2.4*	3.6 - 5.1
BUN (mg/dL)	37*	6.0 - 24
Creatinine (mg/dL)	2.9*	0.5 - 1
Phosphate (mg/dL)	5.1*	2.5 - 4.5
Magnesium (mg/dL)	2.8*	3.5 - 5.3
Uric Acid (mg/dL)	9.1*	3.5 - 7.2
Lactate Dehydrogenase (U/L)	230*	85 - 226
Serum Osmolarity (mOsm/kg)	318*	278 - 305
Hemogram		
White Blood Cell Count (10 * 3/uL)	6.31	3.50 - 10.50
Monocyte (%)	0.5	-
Neutrophil (%)	43.7	-
Lymphocyte (%)	3	-
NRBC (%)	1.4	-
RBC (10 * 6/uL)	4.07	
Hemoglobin (g/dL)	8.1*	12 - 15.5
Hematocrit (%)	26.9*	34.9 - 44.5
MCV (fL)	66.1*	81.6 - 98.3
MCH (pg)	19.9*	27.5 - 33.2
RDW (%)	22.5*	11.8 - 15.6
Platelets (10 * 3/uL)	217	150 - 450

The patient presented with hypercalcemia of malignancy caused by his multiple myeloma, hyperphosphatemia, hyperuricemia, and a new-onset acute kidney injury, as evidenced by the elevation of serum creatinine when compared to baseline. Based on the Cairo-Bishop’s criteria and the lack of a conspicuous trigger that could explain his electrolyte abnormalities, the patient was diagnosed with STLS. Other causes, such as metabolic, medication-induced, granulomatous disease, immobilization, or adrenal insufficiency, were ruled out during the patient’s workup. 

Treatment

The patient was treated with fluid therapy on a continuous infusion with normal saline at a rate of 150 cc/h, with periodic evaluations to monitor electrolyte disequilibrium and fluid overload. Allopurinol 100 mg by mouth once daily and febuxostat 40 mg by mouth once daily were administered for the patient’s hyperuricemia. Treatment for the patient’s hypercalcemia of malignancy included dexamethasone 40 mg by mouth once daily, calcitonin 4 IU/kg subcutaneously every 12 hours for 2 days, and zoledronic acid 4 mg intravenously, single dose. Lastly, the hematologist/oncologist recommended continuing with dexamethasone and initiating treatment solely with bortezomib 2.4 mg subcutaneously once daily once electrolyte imbalances were resolved to minimize the risk of treatment-induced TLS [7].

Outcome and follow-up

The patient’s clinical status improved within a few days. Complete recovery of electrolyte abnormalities was achieved, and the patient was discharged with instructions to follow up with his oncologist the following day. The patient received a total of four doses of dexamethasone and bortezomib, as described above, before seeing the oncologist, at which point lenalidomide 10 mg by mouth once daily was initiated, and the so-called lenalidomide, bortezomib, and dexamethasone (RVD) chemotherapy regimen was established. The patient’s electrolyte imbalance improved on the following visits, and his creatinine level was almost back to baseline.

## Discussion

Multiple myeloma is deemed an indolent malignancy due to its low tumor cell proliferation, where the risk of TLS is exceptionally low (approximately 1%) [8], and its risk of STLS is even lower, with only a limited number of studies documenting similar occurrences [2,9,10]. In contrast to prior findings, however, our clinical vignette highlights a distinctive scenario wherein multiple myeloma stigmata combined with patient- and tumor-specific risk factors played a pivotal role in the development of STLS. 

Potential risk factors linking multiple myeloma with STLS can be grossly dichotomized into tumor- and patient-specific [10-12]. For instance, previous reports have documented that extensive bone marrow involvement and high tumor cell turnover, as evidenced by an increase in lactate dehydrogenase, place patients at higher risk of developing STLS [8-10]. Furthermore, cytogenetic abnormalities, such as translocations or aberrations in chromosomes 11 and 13, deletion of 17p13 causing TP53 deletion, t(4;14) translocation causing FGFR3 overexpression, or monosomy 13, are associated with a higher risk of STLS [13]. On the other hand, patient-specific risk factors include acute or chronic renal impairment, which curtails electrolyte clearance, and advanced age [11].

Our case aligns with previous evidence and illustrates multiple risk factors for STLS. The patient had hypercalcemia in the setting of multiple myeloma and potential myeloma-related kidney injury, which may have exacerbated the impairment of renal function. Additionally, bone marrow biopsy showed high cellularity and abnormal plasma cells, indicating high tumor burden and size. Although our patient lacked any cytogenetic abnormalities, the amalgamation of tumor-specific risk factors, malignant hypercalcemia, and renal impairment collectively contributed to and increased the risk for the onset of STLS.

The diagnosis of STLS is established through the Cairo-Bishop criteria in the absence of recent use of chemotherapy that could have incited electrolyte irregularities [14]. The criteria assess both laboratory abnormalities, such as elevated uric acid, potassium, and phosphate levels, and clinical features, including acute kidney.

In our clinical vignette, the patient presented with hypercalcemia, hyperphosphatemia, hyperuricemia, and acute on chronic kidney injury, as evidenced by an elevation in serum creatinine compared to baseline, all in the context of multiple myeloma. While myeloma-related kidney injury, such as cast nephropathy, may be considered an appropriate differential, it is generally characterized by a chronic course. The acute and subacute onset of the patient’s symptoms, such as new-onset ataxia, significant weakness, and tremors, and satisfaction of the Cairo-Bishop criteria were more consistent with a diagnosis of STLS. This distinction was further reinforced by the absence of identifiable triggers, such as recent chemotherapy, medication use, or other underlying conditions that could explain the rapid metabolic disturbances observed.

The patient’s clinical course further substantiated the diagnosis, as there was rapid improvement following TLS-specific therapy, which included aggressive hydration and the correction of electrolyte imbalances. Such a response is atypical for myeloma-related kidney injury, which generally does not resolve as swiftly with TLS-directed treatments. The early recognition and appropriate management of STLS in this case were critical in preventing further complications.

Contextualizing the entire clinical scenario, we posit that the malignant hypercalcemia germane to the osteolytic lesions of multiple myeloma played a role in exacerbating renal impairment, which may have contributed to the development of laboratory and clinical manifestations consistent with STLS. 

The medical management approach for spontaneous tumor lysis syndrome (STLS) predominantly involves aggressive fluid therapy and correction of electrolyte imbalances to prevent complications [14,15]. Treatment entails administering agents to reduce serum potassium levels and calcium gluconate to stabilize the cardiac membrane, thereby mitigating the potential occurrence of cardiac dysrhythmias [16]. In instances where STLS is established, rasburicase is the preferred treatment over allopurinol. Furthermore, hyperphosphatemia may be rectified through fluid therapy or oral phosphate binders that concomitantly address hypocalcemia. Our patient received all pertinent treatments based on current clinical recommendations, except the incorporation of glucocorticoids and bisphosphonates to suppress osteoclastic activity in response to malignancy-induced hypercalcemia [17].

Our clinical scenario elucidates an unconventional manifestation of hypercalcemia and STLS in individuals afflicted with multiple myeloma, prompting three learning points. Firstly, in multiple myeloma patients with tumor-specific risk factors, hypercalcemia of malignancy may promptly compromise renal function, rendering individuals susceptible to STLS. Secondly, clinicians must exercise awareness regarding tumor and patient-specific risk factors that may warrant continuous monitoring or prophylactic fluid therapy to avoid complications. Lastly, a comprehensive understanding of risk factors, disease presentation, timely diagnosis, and prompt intervention becomes imperative as multiple myeloma can induce a diverse array of electrolyte imbalances, specifically hypercalcemia, and hyperphosphatemia, that complicate the identification of STLS. 

## Conclusions

We presented the case of hypercalcemia and STLS in the setting of multiple myeloma. Although atypical, we posit that the interplay of heightened tumor cell turnover, widespread bone marrow infiltration by plasma cells, and the superimposed acute-on-chronic kidney injury stemming potentially from malignant hypercalcemia and myeloma-related kidney damage collectively predisposed the development of STLS. Our manuscript underscores pivotal observations for clinicians caring for patients with hematological malignancies and renal dysfunction.
